# Utilizing the Brief Child Abuse Potential Inventory in a cohort of children with early childhood caries

**DOI:** 10.1007/s40368-025-01101-x

**Published:** 2025-08-29

**Authors:** Heikki Alapulli, My Blomqvist, Noora Ellonen, Sarimari Tupola, Eeva Nikkola

**Affiliations:** 1 Department of Oral and Maxillofacial Diseases, Helsinki University and Helsinki University Hospital, Helsinki, Finland; 2 Faculty of Social Science, Tampere University, Tampere, Finland; 3 Department of Children and Adolescents, Helsinki University and Helsinki University Hospital, Helsinki, Finland

**Keywords:** Child abuse risk, Risk assessment, Early childhood caries, Dental professionals

## Abstract

**Purpose:**

Dental professionals who regularly interact with child patients are well positioned to identify those at risk of child maltreatment. However, uncertainty surrounding the accuracy of their own observations adds complexity to decision making. The aim of this study was to assess the use of the Brief Child Abuse Potential Inventory (BCAP) in a dental setting among a high-caries child population in Finland.

**Methods:**

Sixty-three parents of children under 5 years of age, who underwent dental treatment under general anaesthesia, completed the BCAP questionnaire as a screening instrument for child abuse risk.

**Results:**

The mean score for abuse risk was 2.84 (SD = 3.37). Notably, 21% of the respondents scored above five, indicating an elevated risk of abuse.

**Conclusion:**

The BCAP exhibits favourable psychometric properties for this population, with the mean abuse score notably higher than that of the general Finnish population. Including this inventory in the dental setting could be helpful for dental professionals, in identifying children at risk of abuse and families who may require additional support.

## Introduction

Dental professionals regularly work with child patients and are in a good position to recognize and report possible child maltreatment (CM) to the relevant authorities. Children may encounter various forms of maltreatment that affect their oral health (Tate et al. 2024). Poor oral health is associated with CM (Duda et al. 2017; Justesen et al. 2025; Keene et al. 2015; Kivistö et al. 2014; Kvist et al. 2018). According to a recent scoping review, child physical abuse and dental neglect were the most commonly identified types of CM in dental settings (Håkstad et al. 2024). However, many studies have found that dental professionals often hesitate to report incidents due to uncertainty about their own observations (Alapulli et al. 2023; Jakobsen et al. 2019; Uldum et al. 2017). If professionals had a reliable tool to support their suspicion, it could lower the threshold for talking about concerns with the families. Eventually, it might also help dental professionals decide when to make the necessary notifications.

The Child Abuse Potential (CAP) Inventory is a caretaker-report measure developed by Professor Joel Milner to estimate the risk of a parent physically abusing their child. It originally consisted of 160 forced-choice (agree/disagree) questions (Milner et al. 1986). It has been adapted, and translated versions have been utilized in several countries (Milner and Crouch 2012). Cross-cultural validation of the Finnish version was published in 2017 (Ellonen et al. 2017). The questionnaire’s length and complex scoring system limit its usefulness, particularly in primary healthcare settings. As a result, a brief version of the CAP Inventory (BCAP) was developed including 24 or 25 of risk items and nine validity items of the original CAP (Ondersma et al. 2005). These items were chosen to reduce the length of the CAP while preserving as much shared variance as possible with the full survey. The goal was to maintain a stable factor structure and useful validity scale, ultimately maximizing the predictive validity of the BCAP (Ellonen et al. 2019; Ondersma et al. 2005). This brief version has been used in several studies conducted worldwide (Abrahamse et al. 2021; Arruabarrena et al. 2022; Lang et al. 2022; Lepistö et al. 2022; McGoron et al. 2024). The validity of the Finnish version in detecting child abuse potential in the general Finnish population has been studied, and the version has displayed favourable properties (Ellonen et al. 2019). There are no previous studies using the inventory in dental healthcare settings.

The aim of this study was to evaluate the application of the Brief Child Abuse Potential Inventory (BCAP) in families whose children were treated under the general anaesthesia (GA) for early childhood caries (ECC). A secondary objective was to identify factors associated with elevated risk of abuse.

## Material and methods

### Study design and participants

This study was conducted at the New Children’s Hospital, Helsinki, Finland, as part of the HECC-CAN-study (ClinicalTrials.gov NCT04898465). The participants in the study were parents who’s up to five years old children were born in Finland and who received treatment for ECC under GA. The parents of children with chronic diseases and special health care needs were excluded from the study.

The BCAP Inventory was conducted as a cross-sectional self-report survey of 89 parents whose children participated in the HECC-CAN study between February 2021 and October 2022 in the New Children’s Hospital, Helsinki, Finland. The Finnish version of the BCAP Inventory was used. Parents who required help from an interpreter were excluded, resulting in 63 parents participating in the study.

### Ethics

This study was approved by the Helsinki University Hospital Research Ethics Board and the hospital administration as part of the HECC-CAN study. Participation was voluntary and was confirmed by signing an informed consent form.

### BCAP inventory

The Finnish version of the BCAP includes 25 items (Table 1) and 9 validity items (Table 2) of the original CAP. The response to each question is given a score of 0 or 1, with a total score varying between 0 and 25. A cutoff score is used to determine the elevated risk, and it can vary by population and context. In the Finnish general population, the abuse scale threshold is set at 5, with 5 or more points indicating a heightened risk of child abuse (Ellonen et al. 2019), which is lower compared to samples from other European countries (Rivas et al 2021).

**Table 1:** The BCAP items and frequencies of items, % (*n*)

Items	Disagree	Agree
I am a happy person	0.0 (0)	100 (63)
Sometimes I feel all alone in the world	90.5 (57)	9.5 (6)
Everything in a home should always be in its place	52.4 (33)	47.6 (30)
I often feel lonely inside	87.3 (55)	12.7 (8)
Children should never disobey	88.9 (56)	11.1 (7)
I sometimes worry that I will not have enough to eat	92.1 (58)	7.9 (5)
People have caused me a lot of pain	71.4 (45)	28.6 (18)
My life is happy	1.6 (1)	98.4 (62)
Children should be quiet and listen^†^	87.3 (55)	11.1 (7)
My family fights a lot	93.7 (59)	6.3 (4)
My family has problems getting along	95.2 (60)	4.8 (3)
I often feel worthless	95.2 (60)	4.8 (3)
Other people have made my life unhappy	84.1 (53)	15.9 (10)
I often feel very upset	88.9 (56)	11.1 (7)
I have a happy life	1.6 (1)	98.4 (62)
I am easily upset by my problems	84.1 (53)	15.9 (10)
I am often depressed	98.4 (62)	1.6 (1)
I am often upset	96.8 (61)	3.2 (2)
A child needs very strict rules	85.7 (54)	14.3 (9)
I am often upset and do not know why	98.4 (62)	1.6 (1)
I often feel very alone^†^	87.3 (55)	11.1 (7)
I often feel alone	92.1 (58)	7.9 (5)
My family has many problems	93.7 (59)	6.3 (4)
Other people have made my life hard	82.5 (52)	17.5 (11)
I sometimes worry that my needs will not be met	69.8 (44)	30.2 (19)

^†^One missing answer

**Table 2:** Frequencies of the BCAP validity scale items, % (*n*)

Items	Disagree	Agree
*Lie scale*		
I sometimes act without thinking^†^	39.7 (25)	58.7 (37)
I sometimes lose my temper	46.0 (29)	54.0 (34)
Sometimes I have bad thoughts	85.7 (54)	14.3 (9)
I sometimes fail to keep all of my promises	46.0 (29)	54.0 (34)
People sometimes take advantage of me	65.1 (41)	34.9 (22)
I sometimes say bad words	60.3 (38)	39.7 (25)
*Random response scale*		
It is okay to let a child stay in dirty diapers for a while	92.1 (58)	7.9 (5)
Children should not learn how to swim	98.4 (62)	1.6 (1)
I know what the right and wrong way to act is	3.2 (2)	96.8 (61)

^†^One missing answer

The validity of the responses was evaluated in the BCAP using a Lie Scale and a Random Response Scale. The Lie Scale included six items and the Random Response Scale three items. Any score of 4 or above on the Lie Scale and 1 or above on the Random Response Scale should be considered invalid, especially when those scores are considered together.

The scores of the inventory were analysed afterwards and were not used for clinical decision making.

### Background data about the families

Descriptive data were gathered from the hospital records and a parental questionnaire (Supplement 1). The sociodemographic characteristics documented via the questionnaire were the respondents’ gender, age, country of birth, number of children in the family, level of education, employment status and family income. The level of education was stratified as low (≤ 12 years) or high (> 12 years) according to the duration of education and the Finnish school system (Ministry of Education and Culture.). The respondents’ own teeth-brushing and smoking habits and their children’s oral health habits were also enquired. Dmft-index and the number of extracted teeth were obtained from the children’s hospital records on the day of operation.

During the hospital visit, one parent from the family completed both the BCAP and the parental questionnaires.

### Statistical analysis

Cronbach’s alpha reliability coefficients were used to describe the internal consistency of the Abuse Risk Scale. Descriptive analyses, namely frequencies and cross tables, were used to describe the data population. SPSS version 29 (SPSS Inc., Chicago, IL, USA) was used for analysis.

Sample size calculation was not performed specifically for this study. The original calculation for the HECC-CAN study was based on the number of children required to detect a difference between the intervention and control groups regarding no-shows after dental care under GA.

## Results

### Elevated abuse risk

In this sample, 4/63 (6.3%) responses were invalid when the Lie and Random Response Scales were considered together. These four respondents were excluded from further analysis.

Among the 59 participants, 12 scored at least 5 on the abuse scale, which translates to 20.6% of respondents with an elevated risk of abuse. The mean abuse score was 2.84 (SD = 3.37) in this population.

### Background characteristics and elevated abuse risk

Among the 59 parents included in this study, 20 individuals (33.9%) were native to Finland. Table 3 presents the sociodemographic data of the respondents categorized according to their elevated abuse risk.

**Table 3:** Sociodemographic data about the respondents

	No risk	At risk	Total
	%(*n*)	%(*n*)	%(*n*)
*Respondent*			
Mother	80.9 (38)	58.3 (7)	76.3 (45)
Father	19.1 (9)	41.7 (5)	23.7 (14)
*Respondent's age*			
25 or less	8.5 (4)	8.3 (1)	8.5 (5)
26–32	34.0 (16)	25.0 (3)	32.2 (19)
33–39	38.3 (18)	33.3 (4)	37.3 (22)
40 or more	19.1 (9)	33.3 (4)	22.0 (13)
*Children in the family*			
1	4.3 (2)	8.3 (1)	5.1 (3)
2	42.6 (20)	41.7 (5)	42.4 (25)
3	27.7 (13)	16.7 (2)	25.4 (15)
4 or more	25.5 (12)	33.3 (4)	27.1 (16)
*Country where born*			
Finland	34.0 (16)	33.3 (4)	33.9 (20)
Other	66.0 (31)	66.7 (8)	66.1 (39)
*Education*			
Low	76.6 (36)	83.3 (10)	78.0 (46)
High	23.4 (11)	16.7 (2)	22.0 (13)
*Family income*			
< 39,999	66.0 (31)	91.7 (11)	71.2 (42)
40,000–79999	23.4 (11)	8.3 (1)	20.3 (12)
> 80,000	10.6 (5)	0.0 (0)	8.5 (5)
*Employment status*			
Employed	46.8 (22)	25.0 (3)	42.4 (25)
Not employed	53.2 (25)	75.0 (9)	57.6 (34)

The mean age of the respondents in the no-risk group was 34.2 years (SD = 5.9) and in the elevated risk group 36.0 years (SD = 8.1). While there was no observed variation in participants' country of origin (Finland or other nations) across the groups, the cohort with elevated risk of abuse contained a higher proportion of parents who were outside the labour market. Furthermore, this group included more families characterized by low income and limited educational attainment.

Table 4 shows the parents' own dental habits as well as their children's caries indices at the time of dental treatment under GA. Parents who brushed their own teeth regularly twice a day were more common in the no-risk group (85.1% vs 14.9%, p = 0.017) than in the high-risk group. The frequency of parental dental visits was not associated with BCAP risk grouping. The average age of the children in both the groups was 3.9 years. Although the mean dmft index and the number of extracted teeth were greater in the elevated risk group, these differences did not reach statistical significance.

**Table 4:** Parents’ oral health habits and their children’s dental health indices

Respondents (parents)	No risk	At risk	Total
	%(*n*)	%(*n*)	%(*n*)
*I smoke*			
Yes	12.8 (6)	41.7 (5)	18.6 (11)
No	87.2 (41)	58.3 (7)	81.4 (48)
*I brush my teeth at least twice a day*			
Yes	85.1 (40)	50.0 (6)	78.0 (46)
No	14.9 (7)	50.0 (6)	22.0 (13)
*I visit a dentist or a dental hygienist annually*^*†*^			
Yes	58.7 (27)	58.3 (7)	58.6 (34)
No	41.3 (19)	41.7 (5)	41.4 (24)

Children	No risk	At risk	Total
	Mean (SD)	Mean (SD)	Mean (SD)
Age	3.87 (0.78)	3.89 (0.87)	3.87 (0.80)
Number of filled teeth (ft)	7.00 (3.32)	7.75 (2.26)	7.15 (3.13)
Number of extracted teeth	1.17 (1.48)	1.25 (1.29)	1.19 (1.43)
Number of decayed missed filled teeth (dmft)	8.17 (3.37)	9.00 (2.70)	8.34 (3.24)

^†^One missing answer

### Reliability and validity

Table 1 presents the frequencies of each BCAP item. The internal reliability of the measure was good, with a Cronbach’s alpha coefficient of 0.825. The variable “I am a happy person” was removed from the reliability scale because of zero variance.

The frequencies of the Lie and Random Response Scales are presented in Table 2.

## Discussion

Identifying the risk of CM is a complex issue for everyone working with the families. In a recent study, Finnish dental professionals identified their uncertainty regarding their own observations of potential CM as the most significant barrier to referring cases to social services (Alapulli et al. 2023). Many risk assessment tools have been developed, mainly for researchers and child welfare professionals, to simplify the accurate and reliable identification of the families at risk (Georgieva et al. 2023; van der Put et al. 2017; Yoon et al. 2021a; Yoon et al. 2021b). For dental settings the tool should be easy to use, time-efficient and suitable for quick screening of possible risk.

Our aim was to evaluate the BCAP Inventory among a sample of parents with young children experiencing poor oral health, as there is a known association between ECC and risk of CM (Folayan et al. 2023; Bahanan and Solafa 2023). To our knowledge, this study is the first attempt to use the BCAP questionnaire in children with ECC.

The mean score for abuse was 2.84 (SD = 3.37), which is higher than the previously reported figure of 1.14 (SD = 1.20) for the Finnish general population (Ellonen et al. 2019). The study of the general population included parents of children who visited various paediatric healthcare facilities, such as primary child healthcare clinics, maternity outpatient clinics and paediatric surgical wards. In that study, the sample consisted of families seeking paediatric healthcare in Finland. Our sample consisted of parents whose children's oral health was compromised and who required dental care under GA. As anticipated, given the established link between poor oral health and adverse childhood experiences identified in previous research (Bahanan and Ayoub 2023; Bradbury-Jones et al. 2021; Toft et al. 2022), as well as between immigration status and higher risk of child abuse (Millett 2016), the mean abuse score in this sample was higher than that reported for the general Finnish population (Ellonen et al. 2019). In the Finnish general population, the cut-off on the abuse scale was 5: five or more points were interpreted as an elevated risk of abuse (Ellonen et al. 2019). Our finding that 20.6% of the respondents had an elevated risk for abuse was also higher than the Finnish general population, where 6% of the respondents had an elevated risk for child abuse.

The validity of the BCAP in this sample was found to be comparable to the previously published study of the general Finnish population (Ellonen et al. 2019), which suggests that this tool may also be applicable in dental practice.

The majority of the parents in our study were immigrants who did not speak native Finnish, but all the children were born in Finland. Parental immigration status and ethnicity have been demonstrated to have a negative impact on the incidence of dental caries in immigrant children, in both their primary and permanent dentition (Rodriguez-Alvarez et al. 2022). Previous research has suggested that parents with a history of migration are significantly more likely to have an invalid protocol in BCAP based on the Lie Scale (Liel et al. 2019). Research also suggests that immigrants often exhibit a reluctance to engage in studies or utilise provided healthcare services, indicating a lack of confidence in these processes (Huslage et al. 2022; Tefera 2024). Our finding that there are similar rates of invalid responses among samples containing an over-representation of immigrants and among the general population is supportive of the use of BCAP among different types of populations, at least in a Finnish context.

In what ways could dental professionals’ benefit from utilising this inventory with families whose children have significant dental issues and possible risk for CM? Dental professionals may find it challenging to enquire about private family matters as this is not typically part of their standard practice. Implementing a new protocol that encourages open and respectful dialogue with parents who score five or more points on potential family difficulties and discussing whether they are seeking or already receiving assistance could offer substantial benefits to both professionals and parents. For instance, parents might not be aware of the preventive child welfare services offered by the municipality or other organizations in the region for children and families who are not clients of child welfare services. There should be seamless opportunities for dental professionals to refer families to preventive social work with minimal barriers. Ideally, dental professionals should collaborate with knowledgeable social workers familiar with the field of dental health care, allowing families to continue to address their needs. In Finland, such arrangements are most common and best available in the Paediatric Dentistry Units of tertiary hospitals.

However, it is important to recognise that risk assessments alone do not determine for example the necessity for child protection notifications. If a dental professional continues to have concerns about the child’s well-being after risk assessment, they should proceed by filing a child welfare report. No individual risk factor alone definitively predicts child maltreatment, but the accumulation of multiple risk factors significantly raises the likelihood of it occurring (Milner and Courch 2017; Yang and Maguire 2018).

It is important to acknowledge the methodological limitations of this study. The sample size of this study was insufficient to demonstrate statistical significance. This study was a component of an intervention study of young Finnish children who underwent dental treatment under GA. During the power analysis phase, we failed to anticipate that a high proportion of immigrant parents would be unable to complete the inventory in Finnish.

## Conclusions

In summary, this study demonstrated that the Finnish version of the BCAP Inventory may serve as a reliable and valid instrument for assessing potential child abuse risk in a group of young Finnish children with high caries rates. The findings also indicated a higher proportion of children at elevated risk of abuse in this group than in a previous Finnish study conducted on the general population. Employing the BCAP as a screening instrument in dental setting could enhance the process of directing families to appropriate interventions.

## Data Availability

No datasets were generated or analysed during the current study.
